# Tissue Is Not the Only Issue in Multimodality, Multidisciplinary Cardiac Mass Evaluation

**DOI:** 10.1016/j.jaccas.2026.106959

**Published:** 2026-03-11

**Authors:** Seyed Hamed Hosseini Dehkordi, Charles B. Porter

**Affiliations:** Division of Cardio-oncology, Department of Cardiovascular Disease, The University of Kansas Health System, Kansas City, Kansas, USA

**Keywords:** cardiac angiosarcoma, multimodality imaging

**Figure:**
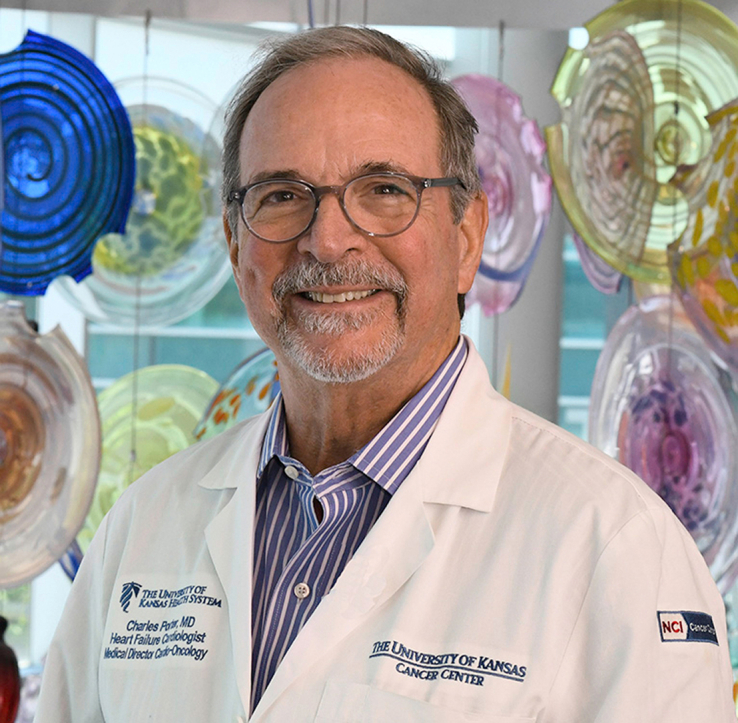

Pons-Riverola et al^1^, in this issue of *JACC: Case Reports*, deserve commendation for their thoughtful and patient-centered management of a complex clinical scenario.^1^ Primary cardiac angiosarcoma is among the rarest and most aggressive malignancies encountered in cardiovascular medicine.^2^ Their case exemplifies the diagnostic, procedural, and therapeutic challenges that can arise when well-established oncologic principles, most notably histopathologic confirmation, collide with prohibitive procedural risks. The authors' multidisciplinary approach involving advanced imaging specialists, cardio-oncologists, cardiac surgeons, interventional cardiologists, and sarcoma oncologists reflects best practice in modern cardio-oncology.

## Primary Cardiac Angiosarcomas

Primary cardiac angiosarcoma is the most common malignant primary cardiac tumor in adults. It typically arises from endothelial cells, most often involves the right atrium, and is characterized by rapid growth, local invasion, and early metastatic spread. Presentation is often nonspecific, ranging from arrhythmias and chest pain to pericardial effusion or tamponade. Diagnosis is usually delayed, and the prognosis is poor, with median survival typically <1 year despite multimodality therapy. Surgical resection, when feasible, offers the best chance for prolonged survival, but complete resection is seldom possible given extensive invasion at presentation. Systemic chemotherapy (taxane- or anthracycline-based) is used in patients with unresectable disease, with evidence largely derived from small series and extrapolated from data on soft-tissue sarcomas.^2^

## Multimodality Imaging as a Diagnostic Surrogate

Even though histopathology remains the gold standard for diagnosis, this case underscores a reality not uncommonly encountered in cardio-oncology: Tissue is not always safely obtainable. American College of Cardiology/American Heart Association guidelines do not directly address this challenge. ESC position statement on cardiac tumors points to multimodality imaging as the foundation of initial evaluation before the final “definitive” tissue diagnosis. It acknowledges that tissue diagnosis is not always possible but stops short of defining pathways for these scenarios.^3^ In this context, the authors demonstrate how advanced imaging, particularly cardiac magnetic resonance (CMR), can function as a diagnostic surrogate rather than merely a triage tool.

CMR's ability to combine anatomical details, tissue characterization, and vascular assessment is uniquely suited for evaluation of suspected cardiac sarcomas. The described findings—heterogeneous T1/T2 signal, marked first-pass perfusion enhancement, and heterogeneous early and late gadolinium enhancement—are highly characteristic of angiosarcoma.^4^ Importantly, demonstration of extensive vascularity across imaging modalities steered the team away from biopsy attempts that carried unacceptable risk.

## Imaging as a Risk-Stratification Tool

An important educational aspect of this case is the use of imaging not only to support the diagnosis but also to specify procedural risk and the chance of success. The coronary angiography revealing tumoral neovascularization from multiple coronary territories, together with CMR perfusion and positron emission tomography-computed tomography findings, not only helped with the diagnosis of the tumor but effectively demonstrated the lack of a biopsy target in the form of an unrecognized noncardiac primary lesion and eventually reframed the clinical question from whether tissue could be obtained to whether tissue should be obtained.

Current position statements provide limited guidance on when empirical therapy is appropriate in the absence of histology. Pragmatic interpretation of these guidelines, however, supports individualized decision-making in life-threatening malignancies when imaging demonstrates classic features and when biopsy risk outweighs the benefit.

As the case is for cardiac myxomas, specific anatomical location and certain morphologic features are characteristic enough to justify a surgical approach for a tentative diagnosis of myxoma, but similar well-defined criteria are not present for all tumors, and this case points to a gap in existing guidance, namely the absence of formalized criteria for “imaging-defined malignancy” in cardiac tumors.

The diagnostic challenge in this case also resembles challenges seen in other areas of cardio-oncology, such as suspected immune checkpoint inhibitor myocarditis, where early treatment is often initiated before or even without definitive “tissue” confirmation.

Future guideline revisions will benefit from incorporating imaging-based “red flag” criteria—extreme vascularity, invasive growth, and characteristic perfusion signatures—that justify empirical therapy in selected cases with multidisciplinary consensus.

## Lessons for the Cardio-Oncologist

This case serves as a reminder for clinicians and advanced cardiac imagers that the goal of diagnosis is not “certainty at all costs” but rather clinical clarity that meaningfully informs management.

Multimodality imaging can provide clarity, even in the absence of tissue.

**Figure 1:**
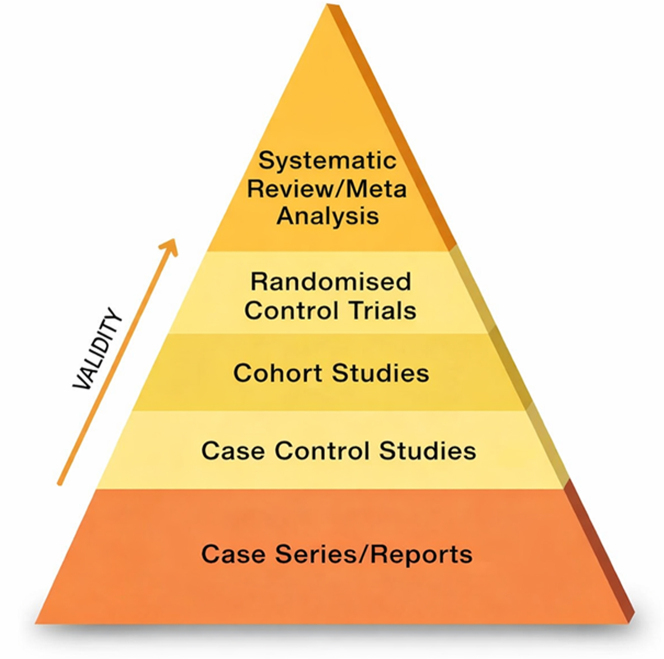
Hierarchy of Evidence Pyramid

Current cardio-oncology practice lies at the intersection of rapidly evolving oncologic therapies in a population with complex cardiovascular comorbidities. As illustrated in Figure 1, traditional evidence hierarchy prioritizes guideline statements supported by multiple randomized trials; however, this framework cannot address many real-world scenarios, as many of these patients are excluded from highly focused randomized trials for these new drugs. In this context, well-described case reports/series, while positioned at the bottom of the evidence pyramid, serve a critical function by identifying novel toxicity patterns, therapeutic trade-offs, and pragmatic decision-making strategies that inform clinical judgment when higher-level evidence is unavailable.

In summary, this case exemplifies the evolving role of multimodality imaging as a cornerstone of decision-making in complex cardio-oncology scenarios. It challenges traditional diagnostic hierarchies while remaining firmly grounded in patient safety, multidisciplinary collaboration, and thoughtful clinical judgment.

## Funding Support and Author Disclosures

The authors have reported that they have no relationships relevant to the contents of this paper to disclose.
